# *Sarcocystis cruzi*: First Molecular Identification from Cattle in Iran

**Published:** 2013

**Authors:** Narges Kalantari, Masomeh Bayani, Salman Ghaffari

**Affiliations:** 1 *Cellular and Molecular Biology Research Center (CMBRC), Babol University of Medical Sciences, Babol, Iran.*; 2 *Department of Laboratory Sciences, Faculty of Para-Medicine, Babol University of Medical Sciences, Babol, Iran.*; 3 *Infectious Diseases and Tropical Medicine Research Center, Babol University of Medical Sciences, Babol, Iran.*; 4 *Department of Parasitology-Mycology, Faculty of Medicine, Babol University of Medical Sciences, Babol, Iran.*

**Keywords:** Cattle, Iran, *Sarcocystis cruzi*, apicomplexa

## Abstract

*Sarcocystis* is a genus of cyst-forming parasites infecting both animals and human. This study aimed to isolate coccidian tissue cysts from muscle of infected animals by a simple method in addition to molecular identification of *Sarcocystis* *cruzi* from the samples. The samples were obtained from commercial source in Babol, Northern Iran. Five grams of calf muscle was cut into 2-3 pieces in 30 ml of phosphate-buffered saline containing 0.1% Tween 80 and homogenized with IKA T25, DIGITAL ULTRA-TURRAX. The homogenate was filtrated twice and used for microscopy examination and molecular analysis. Polymerase chain reaction (PCR) and partial sequence analysis of the 18S ribosomal gene were used to identify the *Sarcocystis* species. Giemsa stain of the filtrated calf muscle samples showed that the sample had ellipse to around tissue cysts contained crescent-shaped bodies. The PCR of the 18SrDNA yielded an 1100 bp DNA band on agarose gel and sequence analysis of the DNA confirmed the isolate as *S. cruzi*. The sequence was deposited in GenBank by Accession No.KC508514. This is the first molecular identification of an isolate of *S. cruzi* in Iran.

*Sarcocystis* species are intracellular protozoan parasites infecting a wide range of vertebrates, even humans. This genus consists of more than two hundred species and is the most prevalent protozoan parasites among domestic animals (1). They have a two-host lifecycle and generally, herbivores and carnivores are intermediate and definitive hosts respectively and omnivores serve the both hosts (2). Different *Sarcocystis* species are assumed to have intermediate host specific relation, e.g., sporocysts of *S. hominis *infect cattle but not pigs while those of *S. suihominis *infect pigs but not cattle (2). However, particular herbivores such as cattle and buffaloes can serve as the intermediate host for numerous *Sarcocystis* species which may infect more than one intermediate hosts. Furthermore, single carnivores e.g. cats and dogs have been identified as competent definitive hosts for a wide range of the parasites (3). Distinguishing of these species has often been performed through morphological characteristic of the Sarcocysts under light or electron microscopy (2, 4-5). This may affect the concrete detection of the species because the appearance of sarcocysts may change in accordance to the location and developmental stage of cysts and other conditions of parasitized cell. Therefore molecular studies have been suggested to confirm morphological species identification (6-8). Also, analysis of DNA sequences is useful to identify *Sarcocystis *species using the variable regions of the 18S rRNA gene which have been reported is a valuable targets for the identification and characterization of different species, even from the same genus (3). In Iran, the prevalence of *Sarcocystis* infections has been reported to be high in domestic animals including cattle, sheep, goats, and camels (9-11) but a few studies have identified the involving species (12).

On the other hand, raw or undercooked meat containing tissue cysts is a source of the *Sarcocystis* and related coccidian cyst-forming infections for humans in addition to carnivores animals. These animals shed large amounts of oocysts infecting human in addition to domestic animals and other herbivores. Therefore, many researchers attempt to isolate tissue cyst- forming coccidian parasites such as *Sarcocystis* spp. and* Toxoplasma gondii* from the meat samples because of several reasons. These reasons include introducing some basic concepts about infectious diseases, cell biology of the parasites, teaching food safety and serological and molecular studies (13). 

In the current study, the first molecular identification of *S. cruzi* from a calf muscle sample in the north of Iran is documented.

## Materials and Methods

**Cyst isolation from skeletal calf muscles**

Muscle samples were obtained from commercial source in Babol, Northern Iran. 5 grams of calf muscle was cut into 2-3 pieces. The sample was added to 30 ml of phosphate-buffered saline containing 0.1% Tween 80(PBS-Tween) pH 7.4(Sigma), in a 50 ml conical flask (14). The resultant was homogenized with IKA T25, DIGITAL ULTRA-TURRAX (Germany) at 8000 RPM for 2 min. The homogenate was then filtered through two layers of gauze twice. The filtrate was collected and saved for further assessments including microscopy examination, cultivation in the laboratory animals and molecular analysis.

**Microscopy examination**

A smear was prepared from the filtrated calf muscle suspension. The smear was air dried and fixed with methanol for 30 second and then stained with Giemsa. The maintenance and concern of laboratory animals complied with the guidelines for the human use of laboratory animals. 2 ml of the filtrate muscle sample was inoculated to four mice peritoneal (0.5 ml per one mouse) (NMRI strain). Two experimentally infected mice were held for one month and two of them were killed with Ethyl ether on day seven. Then, 5 ml sterile PBS was injected to mice peritoneal and then mice peritoneal fluid was collected. The peritoneal fluid was centrifuged 5 minutes at 2000 rpm. The supernatant was discarded and a smear was prepared from the pellet and stained with Giemsa.

**Histology**

Meat sample fixed in 10% formalin were processed as usual, sectioned at 7 µm and stained with haematoxylin and eosin (HE). The sections were examined with a light microscope at 10 and 40ҳ magnifications.

**DNA extraction and PCR analysis**

10 ml of the filtrated sample was centrifuged at 8000 rpm for 5 min. The supernatant was discarded and the pellet was collected. Total DNA was extracted from the pellet using QIAmp1 DNA Mini Kit (Qiagen GmbH, Germany) according to the manufacturer’s instructions. The part of ssurRNA gene was amplified by PCR by a pair of Primers, Primer 1L, Forward, CCA TGC ATG TCT AAG TAT AAG C and Primer 3H, Reverse, GGC AAA TGC TTT CGC AGT AG (BIONEER, Korea) (3). One reaction mixture contained 3 µl of the DNA solution, 20 µl of dH_2_O, 3 µl of 10x PCR buffer (Roche, Germany), 0.5 µl of dNTP (Roche, Germany), 0.5 µl of Tag polymrase (Roche, Germany), 0.5 µl of each primer (20 pmol), 0.5 µl of MgCl _2 _and RNase-free water to make a final volume of 30 µl. The PCR reactions were performed using Thermal Cycler (Bio-Rad, USA) using the following PCR protocol: initial hot start at 93 °C for 2 min, followed by 93 °C for 45 s, 55 °C for 45 s, 72 °C for 2 min; and the final extension at 72 °C for 10 min. PCR products were visualized by UV after electrophoresis on 1% agarose gel stained with Ethidium bromide. DNA was extracted from macro-cysts of *S. moulei* isolated from infected goat sample used as positive control and water were used as negative control. 

Forward primer was used to construct a continuous sequence of the inserted DNA. The continuous sequence was further compared with the previously available *Sarcocystis spp*. sequences of the 18S rDNA in the National Center for Biotechnology Information. The Basic Local Align-ment Search Tool was used to compare with other homologues using CLUSTALW, as implemented by MEGA version 4.0 (15). The *Cryptosporidium hominis* was employed as out-group.

## Results

Five calves muscle samples were obtained.

**Fig. 1:**
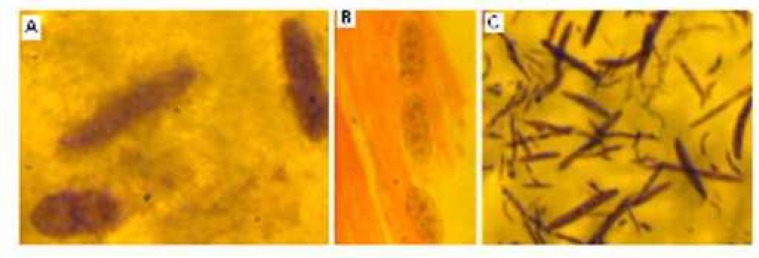
A, *Sarcocystis* tissue cysts in the filtrated of the calf's thigh muscle, stained with Giemsa, at x1000 magnification ; B, *Sarcocystis* tissue cysts in longitudinal sections from thigh muscle of a calf, stained with H&E, at x400 magnification; C, *Sarcocystis* zoites recovered from peritoneal fluid of inoculated mice with the filtrated of the calf's thigh muscle, stained with Giemsa, at x1000 magnification. Images were captured by a JVC-TKCB80 camera (Japan).

Examination of the filtrated calf muscle samples using Giemsa staining method showed that all meat had ellipse to around tissue cysts contained crescent-shaped bodies and some free zoites. The cysts had different sizes (Figure 1.A). Moreover, microscopical cysts were observed in the histological sections of the calves muscle. No inflammatory reaction was found around the cysts in the tissue (Figure 1.B).

**Fig. 2:**
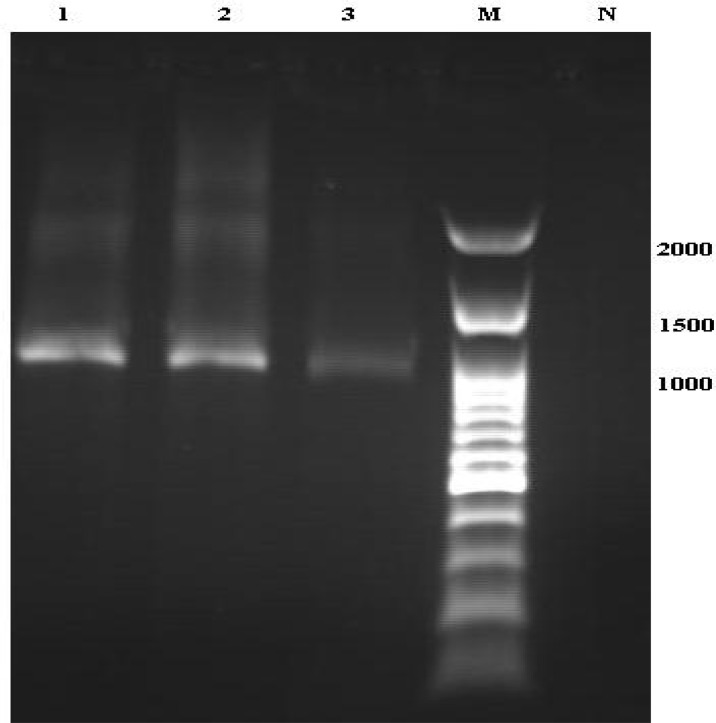
Electrophoresis of PCR product of *Sarcocystis* isolated from goat and calf in northern Iran. Lanes: 1&2,* Sarcocystis* isolated from goat; 3, *Sarcocystis* isolated from calf muscle sample; M, 100 bp ladder DNA size marker; and N, negative control

Furthermore, fresh zoites were seen in the peritoneal fluid of the experimentally infected mice when examined microscopically (Figure 1.C). The recovered zoites had blue cytoplasm and red nucleus and were vary in size. Also, moderate to severe illnesses were progressed in the experimentally infected mice. The illness was gradually relief in the both mice those hold for one month. In addition to the microscopy examinations, PCR analysis was performed to confirm isolated parasite belong to *Sarcocystis* genus. The PCR reaction, using a pair of specific primers, yielded an expected band on agarose gel (~1100 bp) (Figure 2). The assembling of DNA sequence yielded a fragment containing 944 consensus nucleotides. The obtained DNA sequence was analyzed using BLAST software and the isolate was identified as *S. cruzi*. The nucleotide sequence of this parasite was deposited in the GenBank/EMBL/DDBJ database under Accession No. KC508514. Also, bradyzoites obtained from macro-cysts isolated from goat meat sample was used as positive control.

The nucleotide sequence of this parasite previously analyzed by BLAST software and identified as *S. moulei *(Accession No. KC508513).

## Discussion

Cyst - forming coccidian parasites such as *Sarcocystis* spp. has ubiquitous distribution globally. One of important potential source of these infections is meat. Many researchers have been focused on isolation of the tissue cysts as a means to teach food safety, to introduce some basic concepts about infectious disease and cell biology (13). Several methods have been developed to isolate the cysts-forming parasites. The first report on separation of *T. gondii *muscular cyst was in 1960. In this method, a solution containing acid and pepsin was used to recover *T. gondii *from muscular tissues but numbers of infective organisms were too low (16). In subsequent assays Percoll gradient (17) and Dextran (18) were used to obtain more cysts and viable bradyzoites. These methods are relatively labor-intensive and need several materials. In the current study a method was developed to separate coccidian tissue cysts-forming parasites from skeletal muscles of domestic animals using a homogenizer. Generally, this apparatus has been used for batch homogenizing of cell tissues, emulsifying suspen-ded solids, and air pollution filter extraction in food and bio- product processing. The instrument was used for different applications in our laboratory and we tried it for isolation of the cyst and bradyzoites from meat samples. Findings of Giemsa staining method demonstrated that micro-cysts and bradyz-oites of a cyst forming parasite in a drop of homogenized specimen. The same results obtained after filtration of the homogenized sample. In fact, numerous of tissue cysts and viable bradyzoites were obtained without too much squashing of the meat sample. In addition, these obtained through a simple procedure without centrifugation and complicated techniques. In addition to Giemsa stain, the filtrated specimen was injected to mice peritoneal cavity and fresh zoites were recovered which supposed to be a *Sarcocystis* spp.

**Fig. 3:**
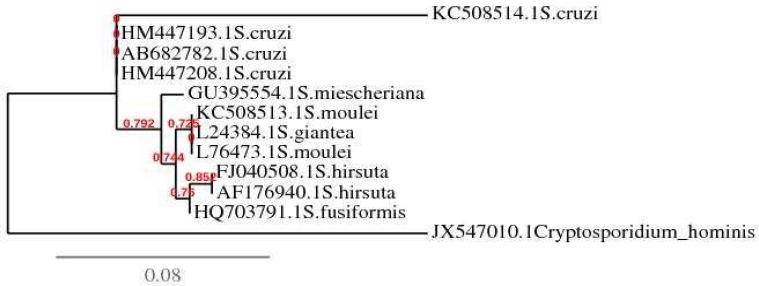
Genetic connection of *Sarcocystis cruzi* isolated from calf muscle sample with other *Sarcocystis* species deposited in the GenBank based on partial 18S rDNA sequence data using http://phylogeny.lirmm.fr

Furthermore, microscopical cysts of *Sarcocy-stis* spp. were observed by direct examination of the calves muscle using a light microscope.

However, the 18S ribosomal DNA region, a suitable target to differentiate the *Sarcocystis* spp., was also confirmed that the isolated parasite belongs to *Sarcocystis* genus (3, 7-8). Since species identification in *Sarcocystis* genus has been reported to be important because some species are more pathogen for certain livestock and are different in infectivity for human. Therefore, sequence analyzing of 18SrRNA gene was performed and compared with the key reference sequences of *Sarcocystis* species deposited in the GenBank which identified that this sequence had 92 % and 93% homology to *S. cruzi* from Japan with Accession No. AB682782 and *S. cruzi* from China with Accession No. HM447208, respectively. The nucleotide sequence of *S. cruzi* obtained inthe present study was deposited in the GenBank/EMBL/DDBJ database under Accession No.KC508514. The phylogenetic analysis using http://phylogeny.lirmm.fr revealed that the sequences of *S. cruzi* from the calf muscle and those from Japan and China from cattle and water buffalo formed a cluster (Figure 3). The reasonable polymorphisms in the ssurRNA sequence between the Iranian *S. cruzi* described in the current study and the other published sequences from Japan and China may be related to geographical isolation. *Sarcocystis* have been reported in livestock from different regions of Iran. Most studies reporting prevalence of infection and there are few have identified *Sarcocystis *species. *S. gigantea* and *S. arieticanis* were detected in slaughtered sheep by PCR-RFLP based on 18S rDNA (11). Recently *S. miescheriana* was reported from a wild boar (12) and *S. fusiformis* was identified in water buffalo (19). But, there is only one report of *S. cruzi* from Iran which identified in two infected calves based on clinical, morphological and pathological criteria after experimental infection in two puppies and consequently in a calf (20). However, *S. cruzi* has a global distribution and mainly reported from cattle and water buffalos of different countries throughout the world (2, 21).

In conclusion, the method described in the current study would be useful in separation of tissue cysts of coccidian parasites from meat to perform further examinations such as molecular characteri-zation of the *Sarcocystis *species from domestic and wild animals.
